# Evaluation of parents’ attitudes and practices related to antibiotic use for their children in Kosovo: a cross-sectional survey

**DOI:** 10.1186/s40545-023-00676-4

**Published:** 2023-12-13

**Authors:** Miradije Imeri, Shaip Krasniqi, Lul Raka, Isme Humolli, Kreshnik Hoti, Zana Imeri, Valbona Zhjeqi

**Affiliations:** 1 Hospital and University Clinical Service of Kosova, 10000 Prishtina, Republic of Kosovo; 2 Faculty of Medicine, University of Prishtina “Hasan Prishtina” (UPHP), Rr. Bulevardi i Dëshmorëve, 10000 Prishtina, Republic of Kosovo; 3 Institute of Public Health of Kosova, 10000 Prishtina, Republic of Kosovo

**Keywords:** Antimicrobials, Parents, Kosovo, Children, Pediatric

## Abstract

**Background:**

Self-medication and lack of patient adherence contribute to antibiotic misuse. This article describes parents’ attitudes and practices regarding use of antibiotics by their children in Kosovo.

**Methods:**

A cross-sectional survey was conducted during data collection. We surveyed a total of 453 parents of children aged 0–15 years, who had experiences with using antibiotics for their children. Correlation tests and regression analysis were used to explore the relationship between variables.

**Results:**

Our findings showed that 42.2% of parents strongly agreed or agreed with the use of antibiotics as a means to cure a cold or flu in their child more quickly. In addition, 29.8% were not aware of antibiotic side effects. Non-compliance with antibiotic treatment was 35.8%, and 28.9% of surveyed parents suggested that they had pressured their pediatricians to prescribe antibiotics for their children. A total of 10.15% of parents had no information on antibiotic resistance, and 34.38% of parents responded that they did not believe that self-medication with antibiotics could lead to resistance. Regression analysis results indicated that gender and age group have a significant influence on the parents’ decision that an antibiotic should be used in children with high fever (*p* < 0.001).

**Conclusions:**

Our findings suggest that antibiotic management by parents in Kosovo is not satisfactory, and more attention should be given to their knowledge of the side effects of antibiotics, bacterial resistance and reduction in the self-medication. Health education, adequate measures and interventions are needed to overcome this situation and ensure rational use of antibiotics in Kosovo.

## Background

The use of antibiotics requires the application of rational prescribing by physicians and adherence to a set of measures and standards by patients to achieve the concept of safe use of antibiotics; otherwise, they can trigger microbial resistance and exert a harmful effect on humans. Antibiotics are not effective in the treatment of viral infections, although the use of antibiotics for these indications is abused in certain cases, especially during self-medication [1]. Antibiotic-resistant infections already cause at least 700,000 deaths annually, worldwide, and this figure could rise to 10 million by 2050 if global action is not taken to prevent bacterial resistance. Approximately 2.4 million people could die between 2015 and 2050 in high-income countries if no sustained efforts are made to prevent antibiotic resistance. [2] There are several factors that contribute to the development of antibiotic resistance. All of these factors can act either directly in bacterial gene resistance or in individual antibiotic efficacy [3]. According to the results of The Central Asian and European Surveillance of Antimicrobial Resistance network (CAESAR), antimicrobial resistance in Kosova is considered a major problem and is characterized by an increase in the incidence of MRSA and Salmonella spp., S. pneumoniae, E. faecalis and E faecium and other bacteria compared to most EU countries [4].

The Ministry of Health of Kosova addressed this health concern initially by approval of the National Strategy and Action Plan to Combat Antimicrobial Resistance 2011–15 [5]. While according to Kosova pharmaceutical legislation antibiotics are prescription-only medicines, the evidence shows that there is a practice of obtaining antibiotics from community pharmacies without a prescription and self-medication is present [6, 7]. Self-medication of antibiotics by humans is considered one of the most influential reasons for the development of antibiotic bacterial resistance [8, 9]. Self-medication is the taking of drugs, on patients or parents own initiative, or on the advice of non-professional individuals, without prescription by medical doctor. It is a worldwide phenomenon that occurs mainly in developing countries, where drug regulation is not fully implemented or drug prescribing is not fully monitored. Self-medication of antibiotics is most prevalent in African countries, South America, Asia and the countries of Southern and Southeastern Europe, including Kosovo [10–12]. Parents practice of self-medication of antibiotics for their children is closely related to their attitudes, health education and their perceptions regarding this group of medicines [13].

Parents influence on antibiotic self-medication for their children is highly valued given the vulnerability of the this population and the potentially harmful effects that antibiotics can exert [14, 15].

The Kosovo health system has not yet reached the full operational level to closely oversee prescribing, including antibiotic prescribing and use. Kosovo's research group is part of several European initiatives that monitor antibiotic use and combat bacterial resistance. According to the survey results conducted through these initiatives, Kosovo had the highest share of total parenteral consumption of ceftriaxone in Europe in 2013 (53.9 DID) [16]. Another related survey, showed that use of antibiotics in children in Kosovo hospitals is very high compared to most European countries [17]. Moreover, one survey performed at primary health care facility in Kosovo shows that parents’ knowledge, attitude and use of antibiotics in their children is not appropriate [18].

Comparatively, Kosovo has the youngest population among European countries and drug use especially antibiotics use for children, should be closely monitored. The comprehensive analysis of parent’s attitudes regarding use of antibiotics for their children in Kosovo will provide us with valuable insights in relation to what influences in relation to use of antibiotics for their children. Specifically, we aimed to evaluate parents' attitudes, perceptions and adherence to antibiotics for their children, as well as to evaluate self-medication practices.

## Methods

### Study design

The survey was a cross-sectional study conducted through a structured, anonymous questionnaire in primary schools in different localities in Kosovo.

### Questionnaire development and validation

The survey instrument was a modified structured questionnaire, developed and adapted to get answers for study questions [19].

This instrument was designed to collect demographic data, knowledge, attitudes and perception of parents for antibiotic use in their children, First, the questionnaire was validated in a pilot survey with 30 parents and following feedback, we adapted and validated its content. The structure of the questionnaire was divided into a demographics section, a section on parents' knowledge about antibiotics and infections, a section on parents' behaviors regarding antibiotic use and a section on parents' relationship with doctors regarding antibiotic prescribing. Respondents’ level of agreement with given statements in the survey was measured on a 5-point Likert scale: strongly disagree–disagree–unsure–agree–agree–strongly agree. In addition, frequency of antibiotic use was measured as follows: always–most of the time–often–sometimes–never; and amount of use was evaluated based on respondents answering: a lot–enough–not a lot–a little–not at all.

### Setting

Prior the conduction of research, we consulted the patient registers in the family medicine centers, and we found that children of the primary school age and they represent a substantial group of the total number of patients receiving the medical treatment in these medical centers. The survey was conducted in 9 (nine) different municipalities through primary schools located in urban and rural areas of Kosova. Initially, we have contacted the Directorate of Education, in seven regions of Kosovo and presented the request for the conduction of the research. The heads of the Directorate department, selected one school in the city and one rural school from their list of schools (choosing only first even number in the list of schools as means of randomization). After the school selection, we contacted the principals of these schools and we get a schedule of meetings with the parents of the children. Randomly, we appointed a time to visit a school without knowing which class will be selected for this survey. Contact with parents in these schools was established during the regular parents’ meetings with class teachers. The questionnaires were distributed by teachers to parents following introduction and provision information about survey. The teachers distributed the questioners to parents inside of classroom, where they were also collected. The researcher was available to facilitate any potential queries or need for clarification by parents, without any interference on their survey answers. The period of collection of data, was between March and July 2018.

### Participants

We surveyed parents of children who had taken an antibiotic at least once for various reasons. The participants were informed in detail about the nature of the study and importance to obtain accurate answers, regarding their knowledge, perceptions and attitude on the use of antibiotics for their children. We have excluded from survey the parents who had a medical profession. Completion of the questionnaire was anonymous, and participation in the study was on a completely voluntary basis. Participants were also informed about their option to withdraw from the study at any time without having to provide a reason. Participants were informed that their data would be kept confidential, and their consent was obtained to participate in the study. By selecting participants from all communities in the country and including randomly selected parents from urban and rural areas, we aimed to mitigate against a possible risk of bias.

This survey was approved by the ethics committee of the Faculty of Medicine of University of Prishtina. In addition, we obtained permission from the respective education departments of the municipalities across the country to survey the parents.

### Data analysis

Descriptive and comparative statistics were used to analyze the results. We used frequencies and cross tabulations for selected variables, while Chi-square (χ2) and *t* tests were used for other variables. The effects between several variables were analyzed using correlation tests. Regression analysis was conducted to ascertain how key demographic variable predicts parents’ attitudes on antibiotic use for their children. Statistical analyses were conducted using SPSS (version 25.0).

## Results

The survey sample consisted of 453 parents. Of these, 49.2% were mothers and 50.8% were fathers. The average parent age was 39.64 years, with a standard deviation of 6.92. Additional demographic details of our respondents are provided in Table 1.

**Table 1:** Sociodemographic data of parents/comparison by gender (n = 453)

Variables	Total (*n* = 530)						*p* value
	*N*	Primary school (*n*)	Secondary School (*n*)	University degree (*n*)	Post graduate (*n*)		
Gender							
Male	230	70	104	40	9		*χ*^2^ = 7.7606 0.51227
Female	223	46	123	50	11		

	*N*	Age (years) of parents					
		≤ 29	30–39	40–49	≥ 50		
Gender							
Male	230	8	73	124	25		*χ*^2^ = 39.4194 0.00001**
Female	223	18	120	81	4		
			Residence				

Gender		Urban	Rural		
Male	230	109	121		*χ*^2^ = 18.2628 0.000019**
Female	223	150	73		

	*N*	Relationship of parents with Pediatrician				
		Family	Friendship	Only professional		
Gender						
Male	230	47	59	124		*χ*^2^ = 3.0797 0.214418
Female	223	48	42	133		

	*N*	Parent information sources about antibiotic use				Other sources	
		Medical doctor	Electronic media	Internet	Family/Friends		
Gender							
Male	230	224	12	18	18	5	*χ*^2^ = 1.4104 0.84238
Female	223	215	10	23	16	3	

	*N*	Perception of parents for antibiotic use				The use of antibiotics does not increase the bacterial resistance	
		Antibiotic should be used in high temp	Antibiotic should use for influenza	Antibiotic use accelerates the healing of common cold	Antibiotic do not have any side effect		
Gender							
Male	230	91	90	110	41	117	*χ*^2^ = 9.437 0.051058
Female	223	62	94	81	39	134	

*p < 0.05, **p < 001

The data presented in Table 1 suggest that there are significant differences between fathers and mothers in terms of age group and urban or rural residence. We did not find statistically significant differences in terms of level of education, relationship with the pediatrician, resources for obtaining information on the use of antibiotics for their children, and attitudes and perceptions toward the use of antibiotics for their children. In most cases, the parent's source of information about the antibiotics used for their children was the pediatrician. However, approximately one in four parents also used alternative non-medical sources for information on antibiotics. No significant difference was observed in relation to parents’ gender and their perceptions statements implying incorrect use of antibiotics (*χ*^2^ = 9.437 for *p* < 0.05158).

In Table 2, we present results related to parents' attitudes and perceptions toward doctors' prescribing and parents’ use of antibiotics for their children. The percentage of parents who answered that they never preferred doctors to prescribe antibiotics for a runny nose was 51.3%, followed by cough (34%), cold (23.2%) and sore throat (10.8%). In 49% of cases, parents do not use the antibiotic without pediatric prescription, while others tend to self-medicate or follow the pharmacist's recommendations (i.e., without doctors’ prescriptions). Parents' responses suggest that parents follow pediatrician instructions on the use of antibiotics in 64.2% of cases, while others did not follow instructions completely resulting on partial parents’ adherence to antibiotic therapy for their children. It is interesting to note that over 60% of respondents (44.6% always and 17% very often) query the need for antibiotic use when their doctors prescribe antibiotics for their children. Most respondents answered that prescribing antibiotics over the phone is not practiced by their doctors. Only 23.8% of parents described access to health services as excellent. The perception of parents reflects that pediatricians overprescribe antibiotics and they exert a relatively high level of influence on the pediatrician in prescribing of antibiotics for their children. Further details of this section are provided in Table 2.

**Table 2:** Parents' perceptions of health services and health workers regarding antibiotics

Variables	Always (%)	Very Often (%)	Sometimes (%)	Rarely (%)	Never (%)
How often would you prefer the pediatrician to prescribe antibiotics for your child when they have a cold?	11.6	8.2	12.4	44.6	23.2
How often would you prefer your pediatrician to prescribe antibiotics for your child if he has a runny nose?	4.6	4.0	6.8	33.3	51.3
How often do you wish your pediatrician would prescribe antibiotics for your child when they have a sore throat?	12.1	13.7	23.4	40	10.8
How often would you prefer your pediatrician to prescribe antibiotics for your child when they have a cough?	5.6	9.3	14.6	35.8	34.7
How often would you give your child an antibiotic without the advice of a pediatrician, because a pharmacist recommended the antibiotic?	4.2	8.2	12.6	26.0	49.0
When your pediatrician recommends an antibiotic, how often do you ask if it is truly necessary for your children?	44.6	17	17.8	15.7	4.9
How often does your pediatrician recommend antibiotics for your child over the phone?	2.9	3.3	4.0	21.8	68
How often do you follow all the pediatrician’s instructions and advice?	64.2	19.9	6.6	6.6	2.7
How often do you urge your pediatrician to recommend antibiotics even when the microbial diagnosis is not clear?	4.9	4.4	3.5	20.8	66.4
How often does your pediatrician explain your child's illness and whether or not your child should take antibiotics?	47.7	21.4	12.1	12.4	6.4
How often does your pediatrician recommend a throat or nose swab before taking antibiotics?	11	11.9	17.9	38.6	20.6

Variables	Excellent (%)	Very Good (%)	Good (%)	Fair (%)	Poor (%)
How would you describe your access to health services?	23.8	40.6	30.2	4.1	1.3

In Table 3, we have presented results reflecting parents' knowledge and attitudes toward antibiotic therapy in their children. In 33.9% of cases, parents strongly agreed or agreed with the use of antibiotics for fever. A total of 41.3% of parents disagreed or strongly disagreed with the statement that flu or colds accompanied by sore throat should not be treated with antibiotics, whereas 42.2% of parents strongly agreed or agreed with the use of antibiotics as a way for their child to heal quickly from a cold or flu. The results show that nearly a third of parents (i.e., 29.8%) were not aware of antibiotic side effects (i.e., strongly agreed, agreed or were undecided). Only half of parents (55.4%) considered that the effectiveness of antibiotics is reduced and resistance increased when they are used without indication. Most parents (i.e., 74.4%) strongly agreed/agreed that antibiotics are widely used reflecting the aware for prescription of this drugs. It was interesting to note that time to visit the pediatrician/doctor was not a reason for parents choosing to administer an antibiotic to their children without it being prescribed (i.e., 84.3% disagreed or strongly disagreed). Among other results, it is worth noting that most parents (i.e., 67.1%) agreed/strongly agreed that they were not informed about antibiotics, and the majority of them (i.e., 81.9%) were concerned about antibiotic side effects. Further details of this section are provided in Table 3.

**Table 3:** Parents' knowledge and attitudes toward antibiotic use in children

Variables	Strongly Agree (%)	Agree (%)	Undecided (%)	Disagree (%)	Strongly disagree (%)
Antibiotics should be used if the child has a fever	13.9	19.9	12.1	32.0	22.1
Flu or colds accompanied by a sore throat should be treated NOT with antibiotics	13.2	27.4	18.1	29.8	11.5
When a child suffers from flu or a cold, it heals faster if it is first treated with antibiotics	16.1	26.1	16.1	26.9	14.8
Antibiotics have no side effects	4.2	13.5	12.1	38.6	31.6
If antibiotics are used without indication, their effectiveness decreases and the bacterium becomes more and more resistant	21.4	34.0	22.3	13.9	8.4
Antibiotics reduce the complications of an upper respiratory tract infection	20.3	38.0	24.1	13.2	4.4
Do you believe that antibiotics are widely used	35.3	39.1	13.6	6.0	6.0
Would you change your pediatrician, because you think he/she does not recommend antibiotics to your children often enough?	5.7	10.8	12.1	41.2	30.2
Would you use an antibiotic again that you have previously used on your child for the same symptoms?	7.1	17.6	11.3	20.7	43.3
Would you insist that your pediatrician prescribe antibiotic therapy if your child was suffering from an upper respiratory tract infection?	11.5	17.4	21.8	31.6	17.7
How often would you give your child antibiotics, because you do not have time to go to the pediatrician?	4.0	2.6	9.1	19.6	64.7
How often would you give antibiotics to your child without the advice of a pediatrician, because your pediatrician has recommended the same antibiotic for the same symptoms in the past?	6.4	6.6	10.6	26.5	49.9
How often would you give antibiotics to your child without the advice of a pediatrician, because a friend/family member recommended it to you?	2.6	4.2	8.8	21.0	63.4
Do you think you are informed about the correct use of antibiotics?	17.2	49.9	17.0	14.4	1.5
Are you concerned about the possible side effects of antibiotics?	61.1	20.8	11.9	4.2	2.0
Do you agree that you would be unhappy if your pediatrician did not recommend antibiotics to your child in case of an upper respiratory tract infection?	13.7	20.3	21.4	11.0	33.6

The regression analysis shows different independent variables such are gender, age groups and residence impact on dependent variables related to parent’ attitudes and practices related to antibiotic use for their children.

Our results indicate that age groups of parents significantly impacted the access to health care services (*F*=11.120, *R*^2^=0.24) suggesting that the age group predicts access to services (*b*=.155, *p*<0.001). Moreover, the age group of parents carries as significant role (*F*=26.391, *R*^2^= − .012) on parents’ attitudes that antibiotic should be used in children with high temperature (*b*=0.55, *p*<0.001), and gender also has a significant impact in this variable for *p*<0.05 (*F*=6.684, *R*^2^= 0.15, *b*=0.331). Further details from the regression analysis are proved in Table 4.

**Table 4:** Regression analysis of different variables of Parents' knowledge and attitudes toward antibiotic use in children

Regression Relations	Beta Coefficient (b)	*R*^2^	*F*	*p* value	Relation confirmed
GE → AHCS	− 0.49	0.02	1.091	.148	No
AG → AHCS	.155	0.24	11.120	.000	Yes
RE → AHCS	.043	0.00	.000	.492	No
GE → RWPE	.029	0.01	.375	.271	No
AG → RWPE	− .012	0.10	4.784	.015	No
AG → AUHT	0.55	− .235	26.391	.000	Yes
GE → AUHT	0.331	0.15	6.684	.005	Yes
AG → ANSE	0.007	.002	0.812	.184	No
GE → ANSE	.122	.003	1.279	.129	No

*p* < 0.05; *GE* Gender, *AG* Age group, *RE* Residence (Urban/Rural), *AHCS* Access to health care services, *RWPE* Relation with pediatrician, *AUHT* Should antibiotic be used in children with high temperature, *ANSE* Antibiotics does not have a side effect

## Discussion

This study provides insights into parents’ attitudes and practices regarding antibiotic use and administration for their children. Parents' attitudes and practices regarding antibiotic use for their children in Kosovo are characterized by nonadherence to antibiotic treatment (35.8%), pressure on pediatricians to prescribe antibiotics (28.9%) and self-medication of antibiotics by parents, because they do not have time to visit the pediatrician, or the pediatrician has recommended the same antibiotic for the same symptoms in the past for their children or the antibiotic was recommended by friend/family member of parents.

It is worth emphasizing that rural parents self-medication among children in Peru was reported to be significantly higher (52% of children received over-the-counter antibiotics) [20] than in Kosova, at 12.4%, while almost matching the results of the surveys in Greece and Italy (10% and 10.4% of parents, respectively, confirm the use of antibiotics for their children without a doctor's prescription) [21, 22]. Chinnasami et al. report that 30% of parents in India confirm the practice of self-medication of antibiotics [23].

Our findings related to parents’ influence on pediatricians to prescribing antibiotics for their children is higher compared to the results of the survey of parents in Turkey (12.4% vs. 6.3%). In addition, a total of 41% of parents in Kosova would recommend an antibiotic for influenza, compared to 38.45% in Turkey [24].

In our study, a substantial number of parents were not aware of antibiotic side effects (29.8%). In comparison studies reported that 26.4% of parents in India [25] and 23.6% in the Philippines considered that antibiotics do not have side effects. [26] These findings highlight a worldwide need for educational campaigns among parents.

The results of the survey conducted in Dhaka revealed that 25% of parents disagreed that antibiotic resistance is a result of unnecessary use of antibiotics, and 10.15% of parents had no idea about the concept of antibiotic resistance. Furthermore, 34.38% of parents answered that they did not believe that self-medication with antibiotics can lead to resistance [27]. The results of a survey conducted in Kosovo showed that 44.6% of respondents had no information about antibiotic resistance and the impact of nonrational antibiotic use. A comparison of the results of our survey with a survey in Tamil Nadu shows that parents in Kosovo answered that sore throats and flu should not be treated with antibiotics in 40.6% of cases, compared to 17.1% in Tamil Nadu [23].

Our results suggest that parental attitudes toward antibiotics in Kosovo are characterized by low adherence to treatment and increased influence on pediatricians for antibiotic prescribing. These findings are consistent with the conclusions of a survey in Spain conducted by Souto-López et al. [28]

Parents’ age group of parents has a significant impact on parent’s access to health care services. Demographic and socioeconomic data such as age and urban or rural status between parents in relation to antibiotic use attitudes and practices differ significantly from each other, while there were no differences in relation to educational level. These results are not consistent with some other studies that found that mothers were more likely to receive antibiotics for their children than fathers because of their emotional relationship with their children [25, 29, 30].

We assume that this finding should explore by other surveys for analyzing of variables with highest impact in the behavior about antibiotic use in the regard of parent’s gender in Kosova [25, 30, 31].

Results from regression analysis show, that gender and age group have a significant impact on parents’ perception that antibiotic should be used in children with high temperature. Our results indicate that 33.77% of parents believed that antibiotic should be used in children with high temperature, while 19% of parents in Saudi Arabia confirmed that antibiotics should be given to children with a fever [4].

Parents' attitudes toward antibiotic therapy in children indicate a moderate level of knowledge and do not reach an optimal and rational awareness level regarding antibiotic indications and conditions of use in the presence of various clinical conditions, such as fever, cold, flu or respiratory infections. Of concern is the insufficient knowledge of parents about the safety of antibiotics and the tendency to self-medicate with antibiotics for their children. All these research findings point to an insufficient level of health education of parents in Kosovo regarding antibiotics. Our findings raise the need for educational awareness campaigns for parents in Kosovo focused on the rational use of antibiotic medications. The findings of this survey could help us better understand and mitigate the unnecessary use of antibiotics in the future.

## Limitations

The collection of data was based on a self-administered questionnaire distributed in schools in different regions of the country during the regular meetings of parents, and the data might be subject to recall bias considering that it would have been better to survey or interview the parents, while they were using antibiotics on their children and not based on previous experience. In this regard, this recall bias as well as the potential lack of representativeness of the study would have been better mitigated if questionnaires were distributed in community pharmacies, i.e., to parents using antibiotics for their children. In community pharmacies, parents present the current behavior and practices regarding the use of antibiotics in their children. However, logistical issues and potential influence of pharmacist in community pharmacies prevented us from doing this. This is considering the fact that our e questionnaire contained some questions related to role of pharmacist and their impact in the selection of antibiotics of parents for their children. The distribution of the parents in the sample size with postgraduate education level was low, and we have not analyzed the marital status of parents, health status and number of children, which may be covariates with the findings of our survey. These demographic components may have affected the findings of our study.

## Conclusions

This study indicates that parents in Kosovo confirm their pediatrician as main source of information for antibiotic use for their children. Self-medication of antibiotics of parents for their children due to different reasons still remains and inappropriate level of knowledge and health education about rational use of antibiotics might influence the tendency for easy access to antibiotics for self-medication. Health education, adequate measures and interventions are needed to overcome this situation and ensure rational use of antibiotics in Kosovo.

## Data Availability

The data are available in hard copy in the form of questionaries and can be audited and inspected at any time by request of competent authorities.

## Abbreviation

DID: DDD/1000 inhabitants per day

## References

[1] WHO. Antimicrobial resistance global report on surveillance. WHO. Published online 2019.

[2] Mark Wetzler L, Pietrocola G, Ellebedy A, Tagliabue Aldo A, Tagliabue A, Rappuoli R. Article 1068 1 Citation: Tagliabue A and Rappuoli R (2018) Changing priorities in vaccinology: antibiotic resistance moving to the top. Front Immunol. 2018;9:1068. 10.3389/fimmu.2018.01068 10.3389/fimmu.2018.01068 PMC5992407 29910799

[3] Levy SB (2002). Factors impacting on the problem of antibiotic resistance. J Antimicrob Chemother.

[4] World Health Organization. Central Asian and Eastern European Surveillance of Antimicrobial Resistance: Annual report 2018. 2020. https://scholar.google.com/scholar?hl=en&as_sdt=0%2C5&q=World+Health+Organization+Central+Asian+and+Eastern+European+Surveillance+of+Antimicrobial+Resistance%3A+Annual+report+2018&btnG. Published online 2020.

[5] Raka L, Kurti A, Jakupi A, Krasniqi S, Turjaka A (2019). Kosovo’s national action plan for antimicrobial resistance. Lancet Infect Dis.

[6] Shabani Z, Mortality KR, Antibiotic self-medication among young adults in Kosovo. Acad Shabani, KJ Redicanmortality, 2018•academia.edu. 2018;ISSN(7):134–140. https://www.academia.edu/download/79038642/ijhms47134-140.pdf. Accessed 13 Nov 2023.

[7] Krasniqi S, Neziri B, Jakupi A (2020). Tuberculosis drug safety and pharmacovigilance in health system of Kosova: a cross-sectional analysis. Pharmacoepidemiol Drug Saf.

[8] Rather IA, Kim B-C, Bajpai VK, Park Y-H. Self-medication and antibiotic resistance: Crisis, current challenges, and prevention. Published online 2017. doi:10.1016/j.sjbs. 2017.01.004 10.1016/j.sjbs.2017.01.004 PMC5415144 28490950

[9] Michael CA, Dominey-Howes D, Labbate M (2014). The antimicrobial resistance crisis: causes, consequences, and management. Front Public Heal.

[10] Wirtz VJ, Herrera-Patino JJ, Santa-Ana-Tellez Y, Dreser A, Elseviers M, Vander Stichele RH (2013). Analysing policy interventions to prohibit over-the-counter antibiotic sales in four Latin American countries. Trop Med Int Health.

[11] Haque M, Rahman NA, McKimm J (2019). Self-medication of antibiotics: investigating practice among university students at the Malaysian National Defence University. Infect Drug Resist..

[12] Lescure D, Paget J, Schellevis F, van Dijk L (2018). Determinants of self-medication with antibiotics in european and anglo-saxon countries: a systematic review of the literature. Front Public Heal.

[13] Ekambi GAE, Ebongue CO, Penda C, Nga EN, Mpondo EM, Moukokoid CEE (2019). Knowledge, practices and attitudes on antibiotics use in Cameroon: Self-medication and prescription survey among children, adolescents and adults in private pharmacies. PLoS ONE.

[14] Simon B, Kazaura M (2020). Prevalence and factors associated with parents self-medicating under-fives with antibiotics in bagamoyo district council, tanzania: a cross-sectional study. Patient Prefer Adherence.

[15] O'Sullivan JW, Harvey RT, Glasziou PP, McCullough A (2016). Written information for patients (or parents of child patients) to reduce the use of antibiotics for acute upper respiratory tract infections in primary care. Cochrane database Syst Rev.

[16] Raka L, Goosens H, Mulliqi G (2015). “Capacity building to implement state of the art surveillance systems for antibiotic consumption and resistance in kosovo”: results of European Union research project in Kosovo. Antimicrob Resist Infect Control.

[17] Krasniqi S, Versporten A, Jakupi A (2019). Antibiotic utilisation in adult and children patients in Kosovo hospitals. Eur J Hosp Pharm.

[18] Bajraktari E, Bajraktari Q. The knowledge, perceptions and behavior of parents regarding antibiotic usage for their children at primary health care institutions in the Republic of Kosovo. *Acad Bajraktari, Q Bajraktariacademia.edu*. Accessed November 13, 2023. https://www.academia.edu/download/62258053/Antibiotic_resistance_in_Kosovo20200302-40446-tmgcqk.pdf. Accessed 13 Nov 2023.

[19] Panagakou SG, Theodoridou MN, Papaevangelou V (2009). Development and assessment of a questionnaire for a descriptive cross - Sectional study concerning parents’ knowledge, attitudes and practices in antibiotic use in Greece. BMC Infect Dis.

[20] Paredes JL, Navarro R, Watanabe T (2022). Knowledge, attitudes and practices of parents towards antibiotic use in rural communities in Peru: a cross-sectional multicentre study. BMC Public Health.

[21] Panagakou SG, Spyridis I, Papaevangelou V (2011). Antibiotic use for upper respiratory tract infections in children: a cross-sectional survey of knowledge, attitudes, and practices (KAP) of parents in Greece. BMC Pediatr.

[22] Pierantoni L, Vecchio A Lo, Parents’ perspective of antibiotic usage in children: a Nationwide survey in Italy. ingentaconnect.com. https://www.ingentaconnect.com/content/wk/inf/2021/00000040/00000010/art00017. Accessed 11 Jan 2023 10.1097/INF.0000000000003221 34437339

[23] Chinnasami B, Sadasivam K, Knowledge, attitude and practice of parents towards antibiotic usage and its resistance. researchgate.net. 2016;3(1):256–261. 10.18203/2349-3291.ijcp20160171

[24] Albayrak A, Karakaş NM, Karahalil B (2021). Evaluation of parental knowledge, attitudes and practices regarding antibiotic use in acute upper respiratory tract infections in children under 18 years of age: a cross-sectional study in Turkey. BMC Pediatr.

[25] Agarwal S, … VY-J of clinical and, 2015 undefined. Antibiotics use and misuse in children: a knowledge, attitude and practice survey of parents in India. *ncbi.nlm.nih.gov*. https://www.ncbi.nlm.nih.gov/pmc/articles/PMC4668498/. Accessed 11 Jan 2023. 10.7860/JCDR/2015/14933.6819 PMC4668498 26674397

[26] Bulario JS, Louise I, Cruz P, Pilapil MC, Gutierrez MM, Bulario S. Factors associated with parental self-medication of antibiotics in Health Centers of Manila. *knepublishing.com*. 2018;2018:891–910. 10.18502/kss.v3i6.2427

[27] Rabbani M, Howlader M, Antimicrobial YK-J of G, 2017 undefined. Detection of multidrug resistant (MDR) bacteria in untreated waste water disposals of hospitals in Dhaka City, Bangladesh. *Elsevier*. https://www.sciencedirect.com/science/article/pii/S2213716517300905. Accessed 12 Jan 2023. 10.1016/j.jgar.2017.04.009 28729208

[28] Cancela OV, Vazquez J, Lopez-Duran A, Figueiras A (2020). Parent-related factors influencing antibiotic use in a paediatric population: a qualitative study in Spain. Wiley Online Libr.

[29] Bert F, Previti C, Calabrese F, Scaioli G, Siliquini R (2022). Antibiotics self medication among children: a systematic review. Antibiotics.

[30] Farha R, Suyagh M, Alsakran L, … MA-TJ of, 2016 undefined. Parental views of antibiotic use in children with upper respiratory tract infections in Jordan. *ajol.info*. https://www.ajol.info/index.php/tjpr/article/view/145220. Accessed 11 Jan 2023.

[31] Hammour K, Al-Saleh S, Integrative WH-EJ of, 2019 undefined. Parental views of antibiotic use in children with upper respiratory tract infections in Dubai. *Elsevier*. https://www.sciencedirect.com/science/article/pii/S1876382019303166. Accessed 11 Jan 2023

